# Spontaneously Ruptured Dermoid Cysts and Their Potential Complications: A Review of the Literature with a Case Report

**DOI:** 10.1155/2020/6591280

**Published:** 2020-03-31

**Authors:** Rebecca Yuan Li, Yogesh Nikam, Supuni Kapurubandara

**Affiliations:** Department of Obstetrics and Gynaecology, Westmead Hospital, Westmead NSW 2145, Australia

## Abstract

Spontaneous ruptures of dermoid cysts are a rare occurrence due to their thick capsules. This is the first systematic review on spontaneously ruptured dermoid cysts. A comprehensive literature search was performed from PubMed, Google Scholar, and MEDLINE. The cases were analysed for patient demographics, presenting signs and symptoms, imaging modalities used, management methods, and outcomes. The majority of cases report an idiopathic cause with symptoms of abdominal pain, distension, and fever. Computed tomography is the most accurate in detecting ruptured dermoid cysts. We also report a case of a 66-year-old who presented with sudden abdominal pain and a low-grade temperature. Imaging showed a 10 cm well-circumscribed hyperechoic mass consistent with a dermoid cyst with no suggestive signs of rupture. She was planned for a laparoscopic bilateral salpingo-oophorectomy. However, intraoperatively, a ruptured dermoid cyst was found with bowel adhesions and chemical peritonitis as cyst contents covered the entirety of the intra-abdominal cavity. Her operative course was complicated by inadvertent iatrogenic small bowel injury, unsuccessful laparoscopy, needing conversion to laparotomy. Despite their benign nature, complications from ruptured dermoid cysts include peritonitis, bowel obstruction, and abscesses. Surgical management by both laparoscopy and laparotomy is successful, with laparotomies more likely to be performed. Complications have mostly no long-term sequelae.

## 1. Background

Dermoid cysts, also known as mature cystic teratomas, are a type of benign germ cell ovarian tumour [1, 2]. They contain well-differentiated tissues that are normally found in other organs including the teeth, hair, skin, fat, muscle, and bone [3]. In premenopausal women who present with an ovarian mass, 70% of those are attributed to a dermoid cyst; however, its true incidence is much lower in postmenopausal women at 20% [1]. The incidence is estimated at 10 per 100,000 women per year [4].

Dermoid cysts are usually incidental findings on medical imaging. They are an insidious tumour where symptoms can appear many years later [3]. Additionally, 10-15% can also present bilaterally [5, 6]. Spontaneous rupture of dermoid cysts is rare, occurring at 1-2% [5, 7].

We report a case report of a spontaneously ruptured dermoid cyst that was managed by laparoscopy initially then converted to a laparotomy due to complications secondary to widespread adhesions and iatrogenic bowel perforation. We also reviewed all cases of preoperative spontaneous rupture of dermoid cysts published in the English literature and set out to identify their distinguishing factors and subtleties from intact dermoid cysts. This is the first systematic review on spontaneously ruptured dermoid cysts. The aim of this review is to summarise and present the patient demographics, cause of rupture, presenting symptoms and signs, imaging findings, and outcomes of surgical management (laparotomy versus laparoscopy).

## 2. Method

A literature search using PubMed MeSH, Google Scholar, and MEDLINE of English articles on the following keywords was performed: spontaneous, dermoid cyst rupture, and ruptured mature teratoma. All cases which reported a widespread rupture into the peritoneal cavity were included. Cases which only reported a localised rupture into surrounding hollow viscus such as the bladder, bowel, and vagina were excluded as they were often described as a fistula or perforation thus making it difficult to complete a comprehensive search to find all those cases. Additionally, these cases are thought to be even rarer than a widespread rupture into the peritoneal cavity [8]. This yielded 87 case reports since 1940 from a total of 74 publications. Prior to this, according to Kistner et al.'s [9] literature review of ruptured dermoid cysts, there have only been 15 reported cases from 1843 to 1938. The results from this review are tabulated below outlining the patient demographics, imaging findings, signs, symptoms, and operative complications associated with spontaneous cyst rupture listed as percentages.

## 3. Case Report

This is a case of a 66-year-old postmenopausal overseas visitor who initially presented to the emergency department with a two-day history of sudden onset sharp abdominal pain in the umbilical and suprapubic regions and a low-grade fever. She is a gravida six, para six, with all vaginal deliveries. She underwent menopause 20 years ago and had no relevant past medical or surgical history.

On examination, she had a temperature of 37.8°C, her pulse rate was 107, and her blood pressure was 107/70. Her respiratory rate was 22, oxygen saturation 97% on room air. Her urinalysis had a trace of leukocytes and protein but was otherwise unremarkable. Her abdomen was soft with rebound tenderness over the lower abdomen. The differential diagnoses included gastroenteritis, diverticulitis, urinary tract infection, or an ovarian cyst with possible intermittent torsion or malignancy. C-reactive protein was elevated at 155 mg/L, and the total white blood cell count was also mildly elevated at 13.3 × 10^9^/L, with a neutrophilia of 11.8 × 10^9^/L.

**Figure 1:**
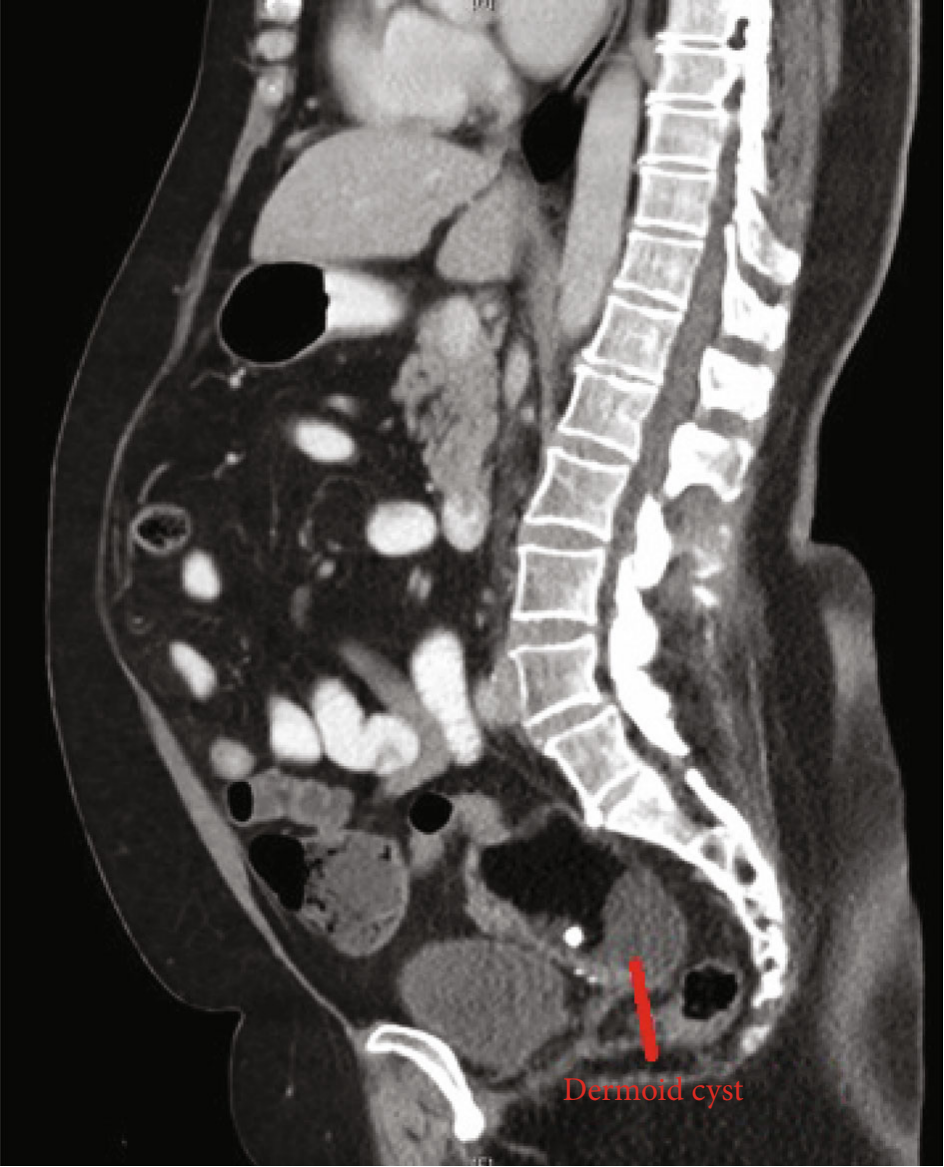
Sagittal view of CT abdo-pelvis showing a likely dermoid cyst in the right adnexa.

**Figure 2:**
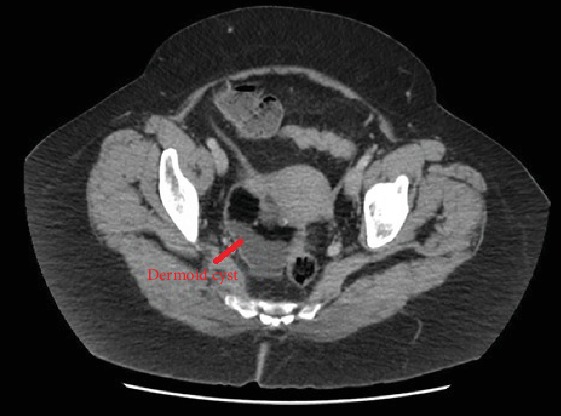
Transverse view of CT abdo-pelvis showing a dermoid cyst in the right adnexa.

She initially underwent a CT abdomen-pelvis which showed a mixed density lesion containing calcifications, fat and fluid density areas in the right adnexa consistent with a 7.4 × 5.6 × 5.9 cm dermoid cyst as seen in Figures 1 and 2. There was no perilesional fat stranding or free fluid identified, and bowel appeared unremarkable. The left adnexa was not mentioned.

**Figure 3:**
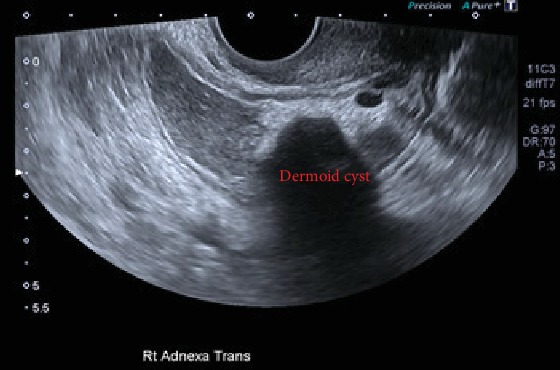
Ultrasound showing a large 10 cm well-circumscribed dermoid cyst.

This was then followed by a pelvic ultrasound which revealed a large right-sided well-circumscribed complex mixed solid cystic mass in the right adnexa measuring 10 × 3.2 × 6 cm as seen in Figure 3. The mass had a hyperechoic component with areas of posterior shadowing and ground-glass internal echoes, with no increased vascularity making the diagnosis most likely consistent with a dermoid cyst although ovarian malignancy could not be excluded. Further investigations revealed CA125 of 29 U/mL.

She was discharged home after the initial investigations with analgesia and planned for a laparoscopic bilateral salpingo-oophorectomy, with the contralateral side removed as risk-reducing surgery as she was postmenopausal. This was as per local hospital policy given the lack of a benefit of keeping the ovaries beyond age of 65 [10]. This occurred eight days postinitial presentation, as this was the earliest elective list available.

**Figure 4:**
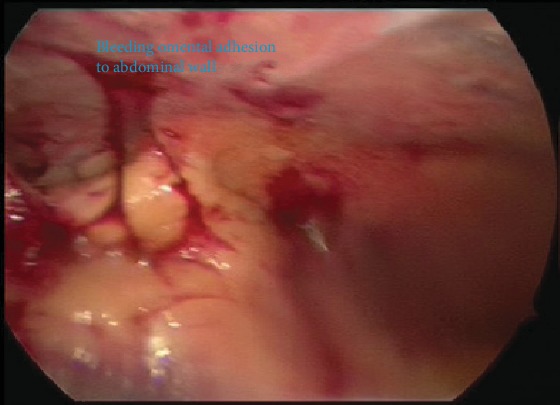
Bleeding omentum adhered to the abdominal wall encountered on attempted laparoscopic entry.

**Figure 5:**
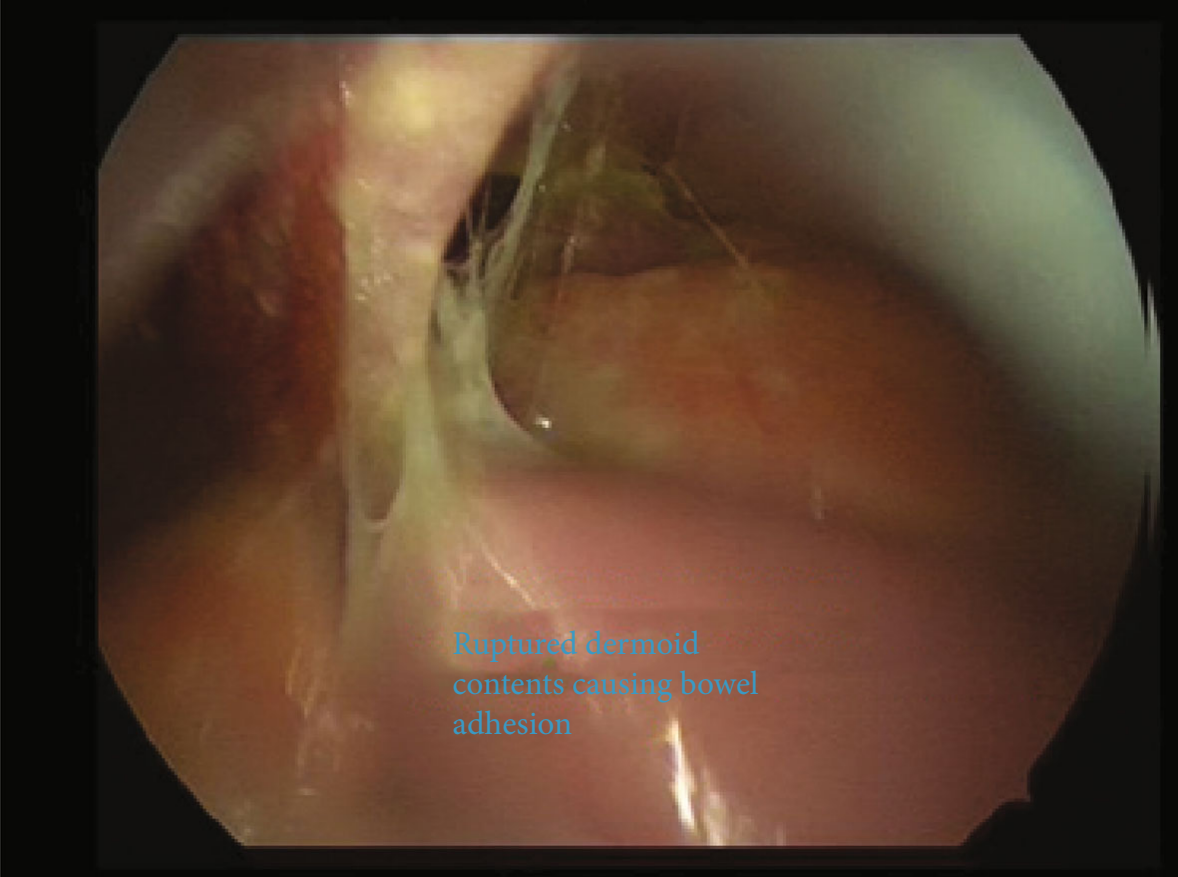
Ruptured dermoid cyst contents at the liver and near the diaphragm.

**Figure 6:**
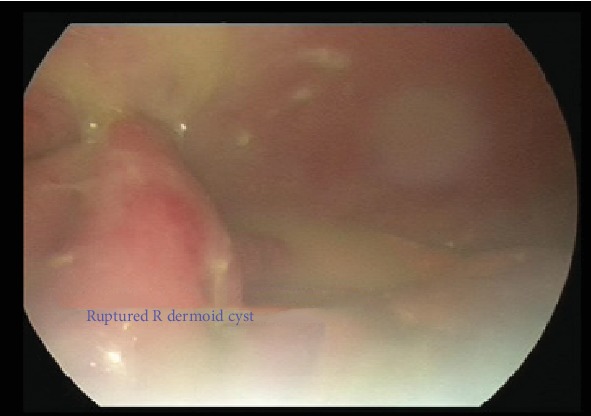
Ruptured right dermoid cyst with cyst content widespread intra-abdominally.

A Veress entry at the umbilicus was first attempted followed by Palmer's Point; however, due to high pressures on insufflation, this entry was abandoned. Direct entry via a 5 mm optical post was then attempted at the umbilicus. There was poor visualisation and bleeding, with a resultant inadvertent small bowel injury as seen in Figure 4. An accessory port was inserted under direct vision at the Palmer's Point, and active bleeding was noted on the omentum adherent to the anterior abdominal wall under the insertion site, with adhesions and cystic contents up to the diaphragm and the liver as seen in Figures 5 and 6, respectively. Thus, the operation was converted to a laparotomy.

Laparotomy findings were of a ruptured dermoid with widespread cystic contents causing multiple bowel adhesions from cystic fluid and adhesions of small bowel to the anterior abdominal wall, a chemical peritonitis, and an iatrogenic small bowel injury of 5 mm × 5 mm. This was managed with a primary repair of enterotomy, bowel run, bilateral salpingo-oophorectomy, vigorous peritoneal irrigation, and adhesiolysis. The skin was closed in a buried running subcuticular suture with 3-0 monocryl.

Her postoperative course was unremarkable with bowel rest to allow the resolution of the chemical peritonitis. She had no complications and was discharged home on the fifth postoperative day. She was followed up in the outpatient gynaecology clinic at six weeks postoperatively as per local hospital protocol. She was well at this point with the wound well-healed and cleared to return home overseas.

The histopathology of the ovarian cyst revealed a mature cystic teratoma. The serosal surface of the bilateral ovaries and tubes showed an intense foreign-body histiocytic reaction including giant cells and hair shaft consistent with a ruptured dermoid cyst. There was no evidence of malignancy seen in any of the histopathology of the dermoid cyst. The abdominal fluid cytology was negative for malignancy and consistent with contents of ruptured dermoid cyst.

## 4. Results

Spontaneously ruptured dermoid cysts occur in a wide range of patients, from 9 to 75 years old, with an average age in this review of 37 years old. The majority occurs in the reproductive age. The reproductive age also accounts for parity as ruptured dermoid cysts were more common in nulliparous women and those that had only 1 or 2 children. Of the cases, 19 (22%) occurred in nulliparous patients, 3 (3%) occurred in grand multiparous patients, and 30 (34%) cases did not mention parity.

80 cases (91%) were of unilateral dermoid cysts, and 8 cases (9%) were bilateral dermoid cysts. In all these cases, the unruptured contralateral cyst was also removed.

## 5. Discussion

Ruptured dermoid cysts most commonly occur in reproductive age women as seen in Table 1. Dermoid cysts have typically and conservatively managed sizes that are small, as they are slow growing at 1.67-1.8 mm/year [2]. When dermoid cysts become symptomatic or are large, particularly over 10 cm, the standard of care for all ages is surgical intervention [2, 11]. There is a higher incidence of coincidental malignancy found in dermoid cysts in postmenopausal women, and radiological and biochemical investigations are not sensitive for malignant dermoid cysts; hence, the recommended standard of care here is surgical removal [12]. Of the ruptured dermoid cysts attributed to malignant transformation, 3 of these were postmenopausal aged patients. When compared to all ruptured dermoid cysts found in the postmenopausal age group, 16% of cases were due to malignant transformation confirming its higher prevalence.

The main cause of rupture, like in this case, is idiopathic. In cases of torsion causing ruptured dermoid cysts, over half (4 cases, 67%) of these are also in conjunction with pregnancy, potentially from changes to position of ovaries and increased vascularity. The sizes of cysts that underwent torsion and subsequent here rupture range from 8 to 22 cm, indicating that the increasing mobility of enlarged dermoid cysts allows for it to move out of the pelvis [15]. Torsion can be involved in prepubescent girls as young as 9 years old up to 31 years old which suggests that torsion is not age-specific, but predominantly in younger age groups [16]. The average size of a torted and ruptured dermoid cyst is 15.8 cm, allowing for the conclusion that torsion has a higher risk in mature ovarian teratomas that are very large or giant (defined as over 15 cm in size) [17]. One particular case of note is a case torsion with ruptured dermoid cyst occurring 20 days after an appendicectomy [18].

**Figure 7:**
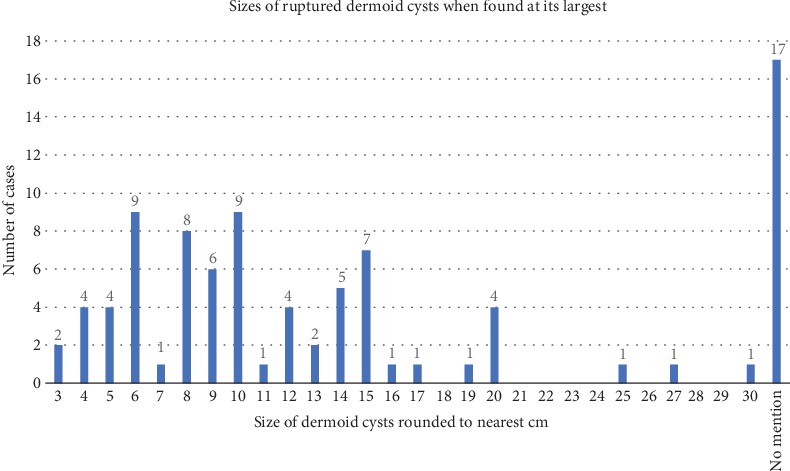
Bar graph showing the number of cases by the size of the ruptured dermoid cyst detected at its earliest either as a surgical finding or imaging finding rounded to the nearest centimeter. It includes one reported case of bilateral rupture where the cysts on both sides were reported as rupture with their sizes. Other cases of bilateral dermoid cyst usually found a unilateral dermoid cyst rupture or did not mention the contralateral cyst.

The size of dermoid cysts can contribute to its predictability for rupture. The majority of ruptured dermoid cysts were found to be in the intermediate size of 6 cm to 10 cm as seen in Figure 7, with a range of 3 cm to 30 cm and an average 11 cm. Large and giant ruptured dermoid cysts are not as commonly found compared to those of intermediate size. This may be due to the surgical management of unruptured dermoid cysts before they enlarge to prevent the complications of spontaneous rupture, which is a standard of care particularly in women of reproductive age [2].

Medical imaging has difficulty in detecting a ruptured dermoid cyst, particularly at the time of rupture. This is especially the case when rupture signs may be subtle as a small cyst wall discontinuity or insidious leakage of cyst contents [19, 20]. The best and most common imaging modality used to detect unruptured dermoid cysts is a transvaginal ultrasound, with studies showing a 99% specificity and 58% sensitivity [8]. However, the accuracy of ultrasound in detecting signs of rupture is poor. CT scan is highly sensitive to adipose tissue which means it can detect ruptured cyst contents presenting as omental infiltration, perilesional fat stranding, fatty fluid in the peritoneal cavity, and intraperitoneal fat implants around the flat surface of the bowel, liver, omentum, and peritoneum [8, 19, 20]. These are all characteristic features of a ruptured dermoid cyst and chemical peritonitis. This highlights the usefulness and accuracy of CT with 88% of cases showing positive signs of rupture as seen in Table 2.

Although reports have shown that ruptured dermoid cysts can also be managed effectively with laparoscopy, but this review found the majority of spontaneously ruptured dermoid cysts were managed with laparotomies as seen in Table 2. This is partly due to the later development of laparoscopic surgery only becoming widespread in the late 1980s and early 1990s, and this review covers case reports published from 1941 to date. The majority of intraoperative and postoperative complications from ruptured dermoid cysts have also occurred in those surgically managed with laparotomies. This may be that the more complicated cases were planned as a laparotomy rather than laparoscopies, negating the need for conversion to laparotomy. Previous studies of the surgical management of intact dermoid cyst show there is an 11-16% rate of conversion to laparotomy due to cyst size and dense adhesions [21, 22] and a 2% rate of bowel injury [21].

The majority of cases of ruptured dermoid cysts can be managed with both laparoscopy and laparotomy with no complications as seen in Table 3. Only 3 cases (3%) had symptomatic patients with residual chronic granulomatous peritonitis post-operatively requiring oral steroids. In this case, complications of conversion from laparoscopy to laparotomy, chemical peritonitis, and iatrogenic bowel perforation occurred. The chemical peritonitis was managed successfully with copious pelvic irrigation and bowel rest and did not require further treatment to the best of our knowledge. However, there are cases where the cyst contents form implants and nodules making them difficult to remove surgically. Kuo et al. [23] report an asymptomatic case of ruptured dermoid cyst where 4 years postsurgery, there was still residual dermoid implants seen on a CT scan. There was also a case of recurrence of dermoid mass up to 17 years postsurgery with abdominal pain and fever requiring further surgical intervention [24]. In this case, our patient was from overseas and only followed up 6 weeks postsurgery. She was advised that this may occur and is for early reviews if there is any clinical change.

One limitation of this review is that the reported size of ruptured dermoid cysts is reduced from its true size as the cyst contents have spilled into the intra-abdominal cavity. Thus, their preruptured or true sizes are not known and can be much larger than what is reported. Only 8 cases have reported a preruptured cyst size as found on imaging and a measurement with the ruptured and collapsed cyst wall as found intraoperatively and the difference ranges from 1 cm to 9 cm. Additionally, many of the measurements are from imaging modalities and not its size as it was found intraoperatively. This is evident in this case where the CT scan measured the cyst to be 7 cm, but the ultrasound found it to be 10 cm, and its intraoperative size was not measured. Furthermore, cyst torsion and the spillage of cyst contents can cause an inflammatory response on the cyst wall which can make the cyst larger than its true size. Future cases should ideally report the size of the cyst on imaging or intact and the size when found ruptured intraoperatively.

Another limitation is that the use of imaging modality to detect ruptured dermoid cysts was not streamlined. This is seen in the results where not all cases utilised ultrasound, CT, or MRIs. The utilisation of which imaging modality may have been due to local protocols in those cases or that rupture was not suspected. For future cases, ultrasound is ideal and first line to characterise the ovarian cyst, allowing dermoid cysts to be differentiated from haemorrhagic or other cysts. If a ruptured dermoid cyst is suspected, a CT scan should then be performed to look for signs and the extent of rupture to allow for careful surgical planning.

A rare case has reported the resolution of symptoms of a ruptured dermoid cyst without surgery; instead, it was treated with a nonsteroidal anti-inflammatory for pain [26]. In Tejima et al.'s [26] case, the dermoid cyst was 6 cm at its initial presentation with transient symptoms of pyrexia, abdominal pain, nausea, and diarrhoea. The patient presented with a temperature of 38°C and tachypnoeic with a respiratory rate of 32/min. Her abdomen was found to be distended, but there was no guarding, tenderness, or palpable mass. Imaging found a pleural effusion and massive ascites. The patient declined surgical management, and follow-up at 12 months after presentation showed a disappearance of the pleural effusion and ascites with nonsteroidal anti-inflammatories. However, the article does not report repeat imaging to assess the size of the dermoid after this time. Thus, in future cases, if patients are not surgical candidates or decline surgical management, nonsteroidal anti-inflammatories can be trialed as treatment for nonperitonitic ruptured dermoid cysts.

## 6. Conclusion

Most spontaneously ruptured dermoid cysts occur idiopathically, but pregnancy and delivery, torsion, and malignant transformation can also contribute to rupture. Dermoid cysts can occur in women of any age but those occurring in postmenopausal women need to have a high suspicion for malignancy despite a low risk of malignancy from radiological or biochemical investigations and should be treated with urgency. The increasing size of dermoid cysts has an increased risk of torsion, particularly those that are 10 cm or larger. Ruptured dermoid cyst can be detected with careful imaging, and if not seen, then repeat imaging should be considered if symptoms are suspicious prior to operative management. Laparotomy is still the mainstay of surgical management for ruptured dermoid cysts; however, it can be successfully managed with laparoscopy with minimal complications. Vigorous irrigation can prevent against granulomatous peritonitis and long-term sequelae.

## Figures and Tables

**Table 1:** Age, cause of rupture, and primary presenting symptoms presenting in ruptured dermoid cases. Ages of patients are classified into age groups of prepuberty, reproductive age, and postmenopausal as per the average age of menarche and menopause in Australia [13]. Increased intra-abdominal pressure from all stages of pregnancy from uterine expansion to labour and delivery is known to cause pressure on surrounding visceral organs and nearby structures, leading to rupture. Additionally, the postpartum period or posttermination of pregnancy is associated with uterine involution which can disrupt the cyst wall leading to rupture [14].

*Age groups*	Number of cases and percentages
Prepuberty (0-13 years old)	2/88 (2%)
Reproductive age (14-50 years old)	68/88 (77%)
Postmenopausal age (51 years old and older)	19/88 (22%)
*Causes of rupture*	Cases and percentages
Idiopathic	32/88 (49%)
Pregnancy, intrapartum, or postpartum	23/88 (26%)
	1^st^ trimester 1/23 (4%)
	2^nd^ trimester 4/23 (17%)
	3^rd^ trimester 10/23 (43%)
	Intrapartum (including those in 3^rd^ trimester) 7/23 (30%)
	Postpartum 7/23 (30%)
Torsion	6/88 (7%)
Malignant transformation (as per histopathology)	6/88 (7%)
Motor vehicle accidents (MVA)	5/88 (6%)
Infection	5/88 (6%)
Posttermination of pregnancy	4/88 (5%) (range 1^st^ trimester to 19/40)
Fall	2/88 (2%)
Vigorous exercise	1/88 (1%)
*Symptoms*	Cases and percentages
Abdominal pain	65/88 (75%)
Fever	32/88 (36%)
Abdominal distension	31/88 (35%)
Nausea, vomiting	25/88 (28%)
Change in bowel habits (constipation, diarrhea)	16/88 (18%)
Palpable abdominal/pelvic mass	10/88 (11%)
Acute abdomen—severe abdominal pain with rigidity	9/88 (10%)
Weight loss	7/88 (8%)
Shortness of breath	4/88 (5%)
Changes to menstrual cycle—e.g., irregular menses	2/88 (2%)
Loss of appetite	2/88 (2%)
Weight gain	1/88 (1%)
Rectal pain	1/88 (1%)

**Table 2:** Table of imaging modalities and operative modes from ruptured dermoid cyst cases. The number of cases that had positive signs for ruptured dermoid cysts is compared to the total cases using different types of imaging modalities. Some cases used more than one imaging modality; others used none. Three cases did not mention their method of management in terms of laparotomy versus laparoscopy. This includes the current case where a laparoscopy was attempted, but it was converted to a laparotomy so is counted as laparotomy.

Medical imaging modalities showing rupture	Number of cases that found signs of rupture over the total number cases that utilized imaging modality and their percentages	Operative management	Cases and percentages
Computed tomography (CT)	22/25 (88%)	Laparotomy	75/88 (85%) Includes 4 cases of caesarean sections
Magnetic resonance imaging (MRI)	4/8 (50%)	Laparoscopic	10/88 (11%)
Ultrasound	18/37 (49%)	Conservative	1/88 (1%) Patient declined surgical management
X-ray	4/24 (17%)		

**Table 3:** Outcomes of dermoid cyst rupture from the reported cases with comparisons between the method of original management of laparotomy vs laparoscopy. Some cases had more than one complication.

Complications	Cases and percentages	Rates of complications encountered on laparotomy	Rates of complications encountered on laparoscopy
No complications	36/88 (41%)	32	4

Chemical/granulomatous peritonitis can present as either or a combination of (1) a histopathological finding of peritoneal biopsies showing peritonitis, (2) an imaging finding of peritoneal thickening/enhancement or perilesional fat stranding, and (3) a surgical finding of dermoid cyst contents overlying entirety of bowel that was still present despite peritoneal irrigation	29/88 (33%) 3 cases needed oral steroids to resolve	26	3

Ileus/bowel obstruction—a clinical finding where the patient does not pass flatus or an imaging finding of dilated bowel loops	13/88 (15%)	12	1

Iatrogenic intraoperative bowel perforation—a clinical finding encountered in surgery for ruptured dermoid cysts (cases where the dermoid cyst was found to be perforating into the bowel lumen were not included)	4/88 (5%)	3	1, this is from this case that the bowel injury was due to laparoscopic entry

Abscess—an imaging finding of confined collection of suppurative inflammatory material postoperatively after surgery for ruptured dermoid cysts	4/88 (5%)	3, 1 case did not mention management by laparotomy or laparoscopy	0

Haemorrhage—a surgical finding of large amount of blood in intra-abdominal cavity	3/88 (3%)	3	0

Death—causes: (i) from cardiac arrest due to massive internal haemorrhage from ruptured dermoid extending to uterine vessels (ii) severe sepsis leading to cardiac arrest (iii) from other injuries with MVA	3/88 (3%)	2	1

Inflammatory dermoid mass recurrence—an imaging finding of calcified mass with necrotic and inflammatory material	3/88 (3%)	3	0

Intra-abdominal collection—an imaging finding of accumulation of fluid in the peritoneal cavity postoperatively after surgery for ruptured dermoid cysts	3/88 (3%)	1	2

Wound infection—a clinical finding of pus discharge from wound or dehiscence	2/88 (2%)	2	0

Disseminated carcinomatosis (from malignant transformation)—a histopathological finding from biopsies in surgery for ruptured dermoid cysts	1/88 (1%)	0	1

Residual dermoid fat implants—an imaging finding after surgery for dermoid cysts shown as small solid masses with low signal intensity [25]	1/88 (1%)	1	0

## Abbreviations

CT: Computed tomography
MRI: Magnetic resonance imaging

## Supplementary Materials

Supplementary Materials Table: all cases reviewed in this literature review pertaining to dermoid cysts rupturing into the peritoneal cavity.
