# Supplementary material for: Spontaneously Ruptured Dermoid Cysts and Their Potential Complications: A Review of the Literature with a Case Report
Source: Case Rep Obstet Gynecol. 2020 Mar 31;2020:6591280. doi: 10.1155/2020/6591280 (PMC7150697; doi:10.1155/2020/6591280)
Supplement: Supplementary Materials — Table: all cases reviewed in this literature review pertaining to dermoid cysts rupturing into the peritoneal cavity. [file 6591280.f1.pdf]

Author	Age	Size (measured at its biggest, rounded to nearest cm)	Gravidity, Parity, Menopausal status if known	Symptoms	Cause of ruptured dermoid cyst	Management	Complications
1. Piper, 1941	50	12	Post-menopausal	Abdominal pain, mass sensation, weight loss	Fall	Laparotomy	
2. Piper, 1941	60	No mention	Post-menopausal	Abdominal pain, distension, nausea, vomiting, fever, diarrhoea	Fall	Laparotomy	Chronic peritonitis
3. Poole, 1947	64	14	Post-menopausal	Abdominal pain, vomiting, diarrhoea, fevers		Laparotomy	
4. Van Orman and Mautner, 1951	25	25, 5 (Bilateral – does not mention if smaller one was ruptured or not)	G1P1, Pre-menopausal	Abdominal pain, mass, distension, constipation	Postpartum	Laparotomy	Granulomatous peritonitis
5. Quer, 1952	24	8	G1P1, Pre-menopausal	Abdominal pain, distension and vomiting	Immediately postpartum	Laparotomy	
6. Kistner et al, 1952	48	15 (Bilateral, Left 6cm does not mention if this ruptured)	G1P1	Abdominal pain, fevers, jaundice		Laparotomy	Granulomatous peritonitis
7. Kistner et al, 1952	31	8	G3P2, Pre-menopausal	Abdominal pain, nausea, vomiting	32 weeks pregnant, torsion	Laparotomy	Paralytic ileus
8. Stein and Kaye, 1954	31	No mention	G2P2, Pre-menopausal	Pelvic mass, rectal pain, weight loss, low grade fever		Laparotomy	Granulomatous peritonitis, fevers
9. Abitbol, 1959	34	No mention	G2P1, Pre-menopausal	Abdominal pain, distension, fever, vomiting		Laparotomy	Paralytic ileus
10. Abitbol, 1959	45	10	G2P2	Abdominal mass		Laparotomy	
11. Waddell et al, 1961	22	6	G3P2, Pre-menopausal	Abdominal pain, nausea, vomiting, diarrhoea	36 weeks pregnant	Laparotomy	
12. Schaffer et al, 1964	27	6	G3P3, Pre-menopausal	Acute abdomen, fever on day 1 postpartum	Preterm delivery at 32 weeks gestation	Laparotomy	Acute chemical peritonitis
13. Jejurkar et al, 1965	35	13	G0P0, Pre-menopausal	Abdominal pain, vomiting, constipation		Laparotomy	Paralytic ileus, chemical peritonitis
14. Cox and Kitay, 1971	30	15	G3P2, Pre-menopausal	Abdominal pain, nausea, vomiting, fevers	16 weeks pregnant, torsion	Laparotomy	Ileus
15. Harris and Mackrell, 1971	20	14	G1P1, Pre-menopausal	Abdominal distension, abdominal pain, fevers	4 days postpartum	Laparotomy	Chemical peritonitis, ileus, wound seroma
16. Lee and Yeung, 1971	36	No mention	G3P2, Pre-menopausal	Acute abdomen, fevers, vomiting	32 weeks pregnant, infection	Laparotomy and classical caesarean section	Bowel perforation of sigmoid colon, ileus, subphrenic abscess, wound infection

17. Panjota et al, 1975	15	No mention	G1P0, Pre-menopausal	Severe shock and collapse	Labour	Laparotomy	Haemorrhage from liver laceration and rupture of uterine vessels, cardiac arrest during surgery, death
18. Waxman, 1976	41	10, 7 (Bilateral – Left 7cm unruptured, ruptured dermoid cyst intra-operatively was found to be 7cm)	G6P6, Pre-menopausal	Abdominal distension, fever, shortness of breath		Laparotomy	
19. Waxman, 1976	22	15	G0P0, Pre-menopausal	Abdominal mass, distension, pain	Torsion	Laparotomy	Re-admitted with inflammatory dermoid mass recurrence as seen on imaging, ascites which was aspirated
20. Waxman, 1976	53	No mention	G0P0, Post-menopausal	Abdominal pain		Laparotomy	Granulomatous peritonitis
21. Mehra et al, 1976	18	8, 8 (Bilateral – both ruptured)	G1P0, Pre-menopausal	Acute abdomen, nausea, vomiting, fever, vaginal discharge	12 weeks pregnant, infection	Laparotomy	Subphrenic abscess
22. Semchyshyn, 1977	45	15	Pre-menopausal	Abdominal pain, shortness of breath		Laparotomy	
23. Giustini et al, 1978	18	No mention	G1P0, Pre-menopausal	Abdominal pain, fevers	Termination of pregnancy	Laparotomy	
24. Stern, 1981	21	27	G0P0, Pre-menopausal	Abdominal distension, weight loss, loss of appetite, fever, nausea and vomiting, discharge of hair from umbilicus		Laparotomy	Bowel obstruction, Abdominal wall sinus
25. Stern, 1981	29	13, 6 (Bilateral – the smaller one did not rupture)	G1P1, Pre-menopausal	Abdominal pain, distension		Laparotomy	
26. Stern, 1981	74	No mention	G12P6, Post-menopausal	Abdominal pain, distension, fever		Laparotomy	Chemical peritonitis
27. Stern, 1981	41	8	Pre-menopausal	Abdominal pain, fever, diarrhoea		Laparotomy	Ileus, pelvic abscess, then sepsis and death of Day 2 post-operatively from cardiac arrest
28. Longmaid et al, 1983	32	6	G2P1, Pre-menopausal	Back pain, fevers, nausea, vomiting, pre-term labour	28 weeks pregnant	Emergency caesarean section for foetal distress	Chemical peritonitis, ileus
29. Stuart et al, 1983	60	15	Post-menopausal	Abdominal pain, diarrhoea, vomiting, low grade fever		Laparotomy	Dermoid mass recurrence needing second laparotomy, chronic granulomatous peritonitis needing oral steroids for 6 weeks
30. Stuart et al, 1983	47	4	G0P0	Abdominal distension, weight gain		Laparotomy	Granulomatous peritonitis

31. Fukushima et al, 1984	28	20	G3P3, Pre-menopausal	Abdominal distension, mass sensation	3 months postpartum	Laparotomy	
32. Graubard and Koller, 1987	44	16	-	Abdominal pain, distension, fever	Infection – Group B strep, Torulopsis glabrata	Laparotomy	
33. Payne-James and Fitzgibbon, 1987	29	5	P2, Pre-menopausal	Abdominal pain, nausea		Laparotomy	
34. Ferrero et al, 1990	19	No mention	Pre-menopausal	Abdominal pain, haematuria	MVA	Laparotomy	Acute chemical peritonitis
35. Holdsworth et al, 1990	28	11	Pre-menopausal	Abdominal pain		Laparotomy	
36. Kommoss et al, 1990	51	4	G3P3, Post-menopausal	Abdominal pain, mass, fever		Laparotomy	Recurrent dermoid masses 17 years as seen on CT later near liver and another mass perforating into the transverse colon needing second laparotomy with liver resection and 7cm segmental transverse colectomy with end-to-end anastomosis
37. Levine et al, 1992	26	8	Pre-menopausal	Syncope, abdominal pain	MVA	Laparotomy	Haemorrhage from avulsion of the ovarian pedicle
38. Sharma and Pendse, 1992	35	No mention	Pre-menopausal	Abdominal mass		Laparotomy	
39. Sharma and Pendse, 1992	22	No mention	Pre-menopausal	Abdominal pain, distension, constipation, vomiting		Laparotomy	Bowel perforation in terminal ileum from dissection of dermoid adhesions
40. Sharma and Pendse, 1992	52	No mention	-	Abdominal pain, distension, constipation		Laparotomy	Acute abdomen post-op from bowel perforation needing second laparotomy (undetected in first laparotomy for dermoid)
41. Sharma and Pendse, 1992	56	No mention	-	Abdominal pain, distention, vomiting		Laparotomy	
42. Bhatla et al, 1993	45	14	P4, Pre-menopausal	Abdominal distension, pain, change in bowel habits, constipation		Laparotomy	
43. Pal et al, 1996	30	12	Pre-menopausal	Fevers, abdominal pain, distension, vomiting, constipation		Laparotomy	Wound infection with dehiscence requiring secondary suture
44. Stenram, 1997	52	7	P3, Pre-menopausal	Abdominal pain, distension		Laparotomy	Sclerosing peritonitis

45. Ling et al, 1998	23	No mention	Pre-menopausal	Abdominal pain, fevers	Termination of pregnancy	Laparotomy	
46. Fibus, 2000	45	10	G0P0, pre-menopausal	Abdominal pain, irregular menses		Laparotomy	
47. Shukunami et al,2000	32	5	G1P1, Pre-menopausal	Abdominal pain	5 weeks postpartum	Laparotomy	
48. Wang et al, 2000	28	5 (Intra-operatively was found to be 4cm)	G1P0, Pre-menopausal	Acute abdomen	Malignant transformation	Laparoscopy	Post-operative abdominal distension, Intestinal obstruction on CT scan with ascites requiring laparotomy, disseminated carcinomatosis
49. Jaworski et al, 2001	27	9	G1P1, Pre-menopausal	Abdominal pain		Laparotomy	Peritoneal melanosis
50. Kumari et al, 2002	25	8	G1P0, Pre-menopausal	Labour with spontaneous rupture of membranes, abdominal pain	39 weeks pregnant in labour – resulting in emergency caesarean section for elevated foetal scalp lactate	Emergency caesarean section	
51. Uysal et al, 2003	50	6	G0P0	Abdominal pain, nausea, vomiting		No mention	Intra-abdominal abscess
52. Takemori and Nishimura, 2003	69	20	G4P3, post-menopausal	Abdominal distension	Malignant transformation	Laparotomy	
53. Wilberg et al, 2003	50	10	G4P3, Pre-menopausal	Abdominal pain, distension, diarrhoea, vomiting		Laparotomy	Granulomatous peritonitis
54. Lichtenberg, 2004	22	10	G4P3, Pre-menopausal	Abdominal pain, mass, fevers,	12 days post termination of pregnancy, infection	Laparotomy	Pelvic peritonitis
55. Phupong et al, 2004	39	19 (Intra-operatively was found to be 16cm)	G0P0, Pre-menopausal	Abdominal distension, shortness of breath		Laparotomy	Granulomatous peritonitis
56. Suprasert etal, 2004	50	10	G1P1, peri-menopausal	Abdominal distension, weight loss		Laparotomy	
57. Suprasert et al, 2004	53	9, 3 (Bilateral – the smaller one was unruptured)	G4P4, Post-menopausal	Abdominal distension, weight loss, fevers		Laparotomy	Granulomatous peritonitis
58. Moriaty et al, 2005	29	No mention	G2P1, Pre-menopausal	Abdominal pain, PPROM, pre-term labour	31 weeks pregnant	Emergency caesarean section for pathological CTG	Chemical peritonitis
59. Roman et al, 2005	26	10	G3P1, Pre-menopausal	Abdominal pain, nausea, vomiting	31 weeks pregnant, torsion	Laparoscopy	

60. Ansell and Bolton, 2006	38	9 (intra-operatively was found to be 6cm)	G1P0, Pre-menopausal	Abdominal pain, vomiting, pre-term labour	36 weeks pregnant	Emergency caesarean for CTG abnormalities	
61. Nitinarvakarn et al, 2006	41	14	G0P0, Pre-menopausal	Abdominal pain, distension, nausea, vomiting, diarrhoea, fevers		Laparotomy	Chemical peritonitis
62. Banerjee, 2007	28	6	P2, Pre-menopausal	Acute abdomen, diarrhoea, fevers	Infection – pseudomonas	Laparotomy	Chronic granulomatous peritonitis
63. Kimbrell et al, 2007	18	15	Pre-menopausal	Abdominal pain, acute abdomen	MVA	Laparotomy	
64. Koshiba, 2007	58	6	G3P3	Abdominal pain, fever		Laparotomy	Chemical peritonitis requiring oral steroids and azathioprine
65. da Silva, 2008	75	14 (intra-operatively was found to be 13cm)	G1P1, Post-menopausal	Abdominal pain	Malignant transformation	Laparotomy	Chemical peritonitis
66. Wang et al, 2008	39	15	G3P2	Abdominal pain	Malignant transformation	No mention	
67. Candela et al, 2009	9	20	G0P0, Pre-menopausal	Acute abdomen	Torsion, 20 days post appendicectomy	Laparotomy	Haemorrhage
68. Chang and Lin, 2009	17	12	Pre-menopausal	Abdominal pain	Vigorous exercise	Laparotomy	Bowel obstruction as seen on imaging
69. Iwata et al, 2009	9	3	G0P0, Pre-menopausal	Abdominal pain		Laparoscopy	
70. Murtaza et al, 2009	55	8	-	Abdominal pain, fever, constipation, vomiting		Laparotomy	Ileus
71. Hosokawa et al, 2010	52	17 (Intra-operatively was found to be 8cm)	-	Abdominal mass	Malignant transformation	No mention	
72. Tanaka et al, 2011	37	12	Pre-menopausal	Acute abdomen, pain, fevers	12 days postpartum	Laparotomy	Uterine perforation
73. Yang et al, 2011	50	4 (intra-operatively was found to be 3cm)	G3P2	Abdominal pain, nausea, fevers		Laparoscopy	Ascites requiring drainage, chemical peritonitis requiring oral steroids
74. Shankar et al, 2013	25	30	G0P0, Pre-menopausal	Abdominal pain, weight loss, distension, shortness of breath, irregular menses		Laparotomy	Intestinal obstruction – seen clinically
75. Tejima et al, 2013	72	6	Post-menopausal	Fevers, abdominal distension, pain, nausea, diarrhoea		Patient declined surgical management – supportive management	
76. Nader et al, 2014	29	9	G2P2, Pre-menopausal	Abdominal distension	2 months postpartum	Laparoscopy	Chemical peritonitis
77. Erbay et al, 2015	54	10	-	Abdominal pain		Laparotomy	Granulomatous peritonitis

78. Kuo et al, 2015	31	No mention	G1, Pre-menopausal	Abdominal distension, weight loss		Laparotomy	Residual dermoid fat implants in subphrenic and perihepatic regions on CT scan, patient is asymptomatic
79. Joshi et al, 2016	26	9	Pre-menopausal	Acute abdomen, pain, vomiting	Termination of pregnancy	Laparotomy	
80. Lim et al, 2016	32	5	Pre-menopausal	Abdominal pain	15 weeks pregnant	Laparoscopy	
81. Haidar and Sharaf, 2017	45	No mention	Pre-menopausal	Abdominal pain	MVA	Laparoscopy	Died from other injuries from MVA – pneumothorax, lung contusions, liver haematoma, brain contusions, developed pneumonia and renal impairment and subsequent cardiac arrest
82. Yohann et al, 2017	15	4	Pre-menopausal	Abdominal pain	MVA	Laparoscopy	
83. Chaudhary et al, 2018	28	6 (intra-operatively was 5cm)	G2P1, Pre-menopausal	Abdominal pain	24 weeks pregnant	Laparotomy	Chemical peritonitis
84. Aydin et al, 2019	34	3	Pre-menopausal	Abdominal pain, loss of appetite	23 weeks pregnant	Mini- laparotomy at McBurney's point	
85. Chandi et al, 2019	27	20	G2P1, Pre-menopausal	Abdominal pain preterm labour, abdominal distension postpartum	30 weeks pregnant, torsion	Laparotomy	
86. Choi et al, 2019	42	9 (Bilateral – likely both ruptured, but case report not entirely clear)	-	Abdominal pain, distension, fever	Malignant transformation – malignant melanoma	Laparotomy	
87. El Moussaoui et al, 2019	31	6 (Bilateral, other side had 2cm dermoid unruptured)	G0P0, Pre-menopausal	Abdominal pain, distension		Laparoscopy	Chemical peritonitis

Supplementary Table: All cases reviewed in this literature review pertaining to dermoid cysts rupturing into the peritoneal cavity.
